# Evaluation of alternative vaccination routes against paratuberculosis in goats

**DOI:** 10.3389/fvets.2024.1457849

**Published:** 2024-11-27

**Authors:** Miguel Criado, Marta Silva, Noive Arteche-Villasol, David Zapico, Natalia Elguezabal, Elena Molina, José Espinosa, María del Carmen Ferreras, Julio Benavides, Valentín Pérez, Daniel Gutiérrez-Expósito

**Affiliations:** 1 Departamento de Sanidad Animal, Instituto de Ganadería de Montaña (IGM) CSIC-ULE, León, Spain; 2 Departamento de Sanidad Animal, Facultad de Veterinaria, Universidad de León, Campus de Vegazana, León, Spain; 3 Departamento de Sanidad Animal, NEIKER-BRTA, Instituto Vasco de Investigación y Desarrollo Agrario, Derio, Spain

**Keywords:** paratuberculosis, oral vaccine, intradermal vaccine, immunization strategies, goat

## Abstract

Paratuberculosis is a chronic granulomatous enteritis, caused by *Mycobacterium avium* subspecies *paratuberculosis* (*Map*), that affects ruminants worldwide. Vaccination has been considered the most cost-effective method for the control of this disease in infected dairy herds. However, currently available vaccines do not provide complete protection and interfere with the diagnosis of both paratuberculosis and bovine tuberculosis, limiting its use. Because of that, efforts are being made for the development of new vaccines. The primary objective of this study was to evaluate the efficacy of two whole-cell inactivated experimental vaccines against paratuberculosis in goats, administered through the oral (OV) and intradermal (IDV) routes, and compare them with that of the commercial subcutaneous vaccine Gudair^®^ (SCV). Over an 11-month period, the effect of vaccination and a subsequent *Map* challenge on the specific peripheral immune responses and *Map-*DNA fecal shedding were recorded. At the end of the experiment, tissue bacterial load and lesion severity were assessed. The experimental vaccines did not induce specific humoral immune responses and only elicited mild and delayed cellular immune responses. Although both experimental prototypes were not able to reduce fecal shedding, the OV reduced lesion severity, whereas the IDV prototype reduced the tissue bacterial load. Moreover, although the SCV did not confer sterile immunity, it outperformed both experimental vaccines in all these parameters.

## 1 Introduction

Paratuberculosis (PTB) is a chronic granulomatous enteritis produced by *Mycobacterium avium* subspecies *paratuberculosis* (*Map*). Domestic and wild ruminants are the most commonly affected species by this worldwide distributed disease, which is officially recognized by the World Organisation for Animal Health (WOAH) (1), and regulated under the EU Animal Health Law (2). It causes important economic losses in cattle, sheep, and goat livestock industries, where PTB is highly prevalent (3), as seen by the estimated herd-level prevalences in southern Spain, which are 66.3% for sheep and 90% for goats (4). Control programs based on hygiene and management measures are hampered by the ability of *Map* to persist in the environment. In addition, although diagnostic tests are improving, sensible detection of infected animals is still challenging, especially in the early stages of infection. Therefore, vaccination has been considered the most cost-effective control tool in affected herds (5–9), as it reduces the colonization of intestinal tissues, *Map* shedding, and the number of clinically affected animals (10, 11). However, currently available commercial vaccines present several drawbacks, as they do not fully protect against infection or prevent transmission (12), they induce the formation of a large granuloma in the injection site (13), and they could interfere with PTB diagnosis (14–16), and with the official bovine tuberculosis (bTB) diagnostic tests (17). As a result of this interference, vaccination has been prohibited in several countries with eradication programs against PTB (18), and many countries with bTB control programs do not allow vaccination against PTB in cattle with current vaccines (5).

Due to this diagnostic interference, there has been widespread reluctance to adopt vaccination strategies for goats in countries where vaccines are available for this species. Furthermore, the relatively smaller economic importance of goats in developed countries likely contributes to the lower number of vaccination trials conducted for goats than those for cattle and sheep. Nevertheless, goats are highly susceptible to PTB and frequently develop diffuse forms of the disease (5, 19), and in this species, hygiene and management measures are particularly challenging; for example, strategies such as individual testing and culling are prohibitively expensive. Previous efforts relying on these measures have not successfully reduced the incidence of the disease. In contrast, vaccination has been shown to be both highly effective and economically viable (5, 8). However, differences in immune responses between cattle and goats to other vaccines (20) should be considered in developing new vaccines against PTB.

All currently approved vaccines and most which were approved until recently are inactivated and administered subcutaneously. One exception to this was Neoparasec^®^, which used a live-attenuated 316F *Map* strain and was discontinued in 2001. To avoid the drawbacks previously mentioned, efforts have been made in the development of alternative vaccination strategies against PTB over the last decades. Many attenuated vaccines have been studied, although, in addition to the risks associated with this type of vaccine, they share most of the disadvantages of the available inactivated vaccines. One of the most comprehensive recent trials conducted in five different laboratories, evaluated 22 attenuated *Map* strains, finding that none of them constituted an optimal vaccine candidate (21). Two of these attenuated strains have been tested in other *in vivo* studies. A K10 ΔpknG mutant did not protect goats against wild-type K10 and was able to persist viable in the studied tissues, highlighting one of the issues regarding live-attenuated vaccines. On the other hand, a K10 ΔrelA mutant has demonstrated the capacity of eliciting a cytotoxic T-cell response when orally inoculated, and reduced tissue colonization in goats and cattle (22–24). Similarly, an oral live-attenuated MAP A1-157 ΔBacA has been very recently tested in a short-term study in calves, inducing a proinflammatory profile in peripheral PBMCs and reducing *Map* burden in some tissues (25). However, these studies lasted under 3 months after the *Map* challenge, and though a reduction in bacterial load was observed, no vaccinated animal reached sterile immunity. In addition, different recombinant proteins and protein cocktails have failed to offer complete protection in both murine and goat models (26, 27). Well-designed DNA vaccines have similar advantages to subunit vaccines but share limited immunogenicity (28), a few of them, encoding different *Map,* antigens have also been studied in murine and sheep models, offering variable levels of protection (29, 30). To summarize, all the alternatives tested do not outperform inactivated vaccines in terms of protection.

Among other different strategies, alternative to the currently available vaccines, recent studies have explored the combination of different adjuvants (31–33) and non-subcutaneous administration routes (33–35). Regarding the latter, the oral (36–42) and intradermal (43–45) vaccination routes have been studied in mycobacteria species such as *M. bovis* and *M. tuberculosis* mainly in ruminants and several wildlife species, as well as in mice and humans, respectively. The oral route has been proposed to achieve an increased activation of mucosal immunity, as this is the entry site for *Map*. Studies have shown that it may stimulate the peripheral immune response to a lesser extent which could be advantageous as it would not interfere with the diagnosis of PTB by ELISA, and it could also decrease the interference with bTB diagnostic tests (35). Similarly, it has been proven that oral vaccination with inactivated *M. bovis* does not interfere with bTB diagnosis in cattle (46) and goats (39). In contrast, regarding intradermal vaccination, the dermis and epidermis are rich in antigen-presenting cells, and therefore, this delivery route should be more efficient at eliciting immune responses with smaller amounts of antigen (47). This route is widely employed in human tuberculosis vaccination (43–45) and has been shown to be more effective than intramuscular or subcutaneous vaccination against other diseases caused by intracellular pathogens, such as influenza virus and Hepatitis B virus (48, 49). Additionally, intradermal delivery is gaining attention due to the development of microneedle and needle-free injection devices, which decrease the risk of disease transmission, and the injection site lesions eliminate the risk of residual needles and improve animal welfare (43–45, 50).

Regarding experimental trials with non-subcutaneous vaccines against PTB in ruminants, several oral live-attenuated vaccines have failed to protect sheep (51) or goats (21, 34) from infection. Nevertheless, orally administered, whole-cell, inactivated, vaccines have been recently tested in the rabbit model: a non-adjuvanted vaccine (35) and one adjuvanted with Quil A^®^ (33), have been shown to exert protection against *Map* infection. However, to our knowledge, no intradermal PTB vaccine has been tested in ruminants up to date. In rabbits, an inactivated, non-adjuvanted whole-cell intradermal vaccine against *Map* induced a higher bacterial clearance and less cross-reactivity with the tuberculin skin test than Silirum^®^ and was more protective against infection than a similar oral vaccine in rabbits (35).

Based on recent promising results, alternative immunization routes could potentially overcome some of the disadvantages of the currently available subcutaneous PTB vaccines. However, *Map* infection is hard to model (52) due to several factors like *Map* strain (53), passage level (54) host species and breed (55), exposure age (56), and the long incubation period of the disease, all of which have a great influence on disease outcome. Because of this, there is still a lack of research in this field, particularly on ruminants whose susceptibility and immune response against *Map* may differ from that of laboratory animals such as rabbits or mice (21). On this basis, the main objective of the present study was to test the efficacy of two whole-cell, inactivated vaccines against *Map*, administered through the intradermal and oral routes, and compare it with a commercial subcutaneous vaccine in goats (Gudair^®^, CZ Vaccines, Porriño, Spain).

## 2 Materials and methods

### 2.1 Animals and experimental design

A total of 28, 1-month-old goat kids of the Murciano-Granadina breed, randomly selected from a commercial flock without a previous history of PTB or TB were used in this study. Animals were kept in the experimental facilities of the Instituto de Ganadería de Montaña (ULE-CSIC). Before the study, all animals were tested negative for both PTB and tuberculosis, using the PTB antibody ELISA (ID Screen^®^ Paratuberculosis Indirect, IDvet), and the WOAH-validated interferon-*γ* release assay (BOVIGAM^™^ TB Kit) against both avian (PPDa) and bovine (PPDb) protein derivatives (57).

**Figure 1:**
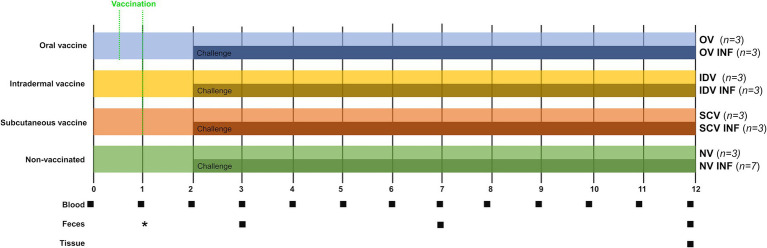
Experimental design scheme. After a 2-week adaptation period, goat kids were divided into four groups and vaccinated through different routes [oral (OV), intradermal (IDV), and subcutaneous (SCV) or left unvaccinated (NV)]. Experimental (OV and IDV) and commercial vaccines (SCV—Gudair^®^) were used. One month after vaccination, three or seven animals from each group were orally challenged with *Map* (INF). A total of eight groups were formed: OV (oral vaccine), OV-INF (oral vaccine, challenged), IDV (intradermal vaccine), IDV-INF (intradermal vaccine, challenged), SCV (subcutaneous vaccine), SCV-INF (subcutaneous vaccine, challenged), NV (non-vaccinated), and NV-INF (non-vaccinated, challenged). Squares indicate sampling time points (blood, feces, and tissues) from the beginning of the study to 12 months. *Fecal samples to assure the vaccine reached the intestine were taken from the orally vaccinated animals at 3, 7, and 14 dpv.

After a 2-week adaptation period, animals were first divided into four groups: oral vaccine (OV) (*n* = 6), intradermal vaccine (IDV) (*n* = 6), subcutaneous vaccine (SCV) (*n* = 6), and non-vaccinated (NV) (*n* = 10) (see Figure 1). Animals of the three first groups were vaccinated using different inoculation routes, and the last group was not vaccinated. Subsequently, 1-month post-vaccination (1 mpv), half of the goats immunized with each different vaccine (*n* = 3), and seven goats of the non-vaccinated group were challenged with *Map* (Figure 1). Before the experimental challenge, the orally vaccinated animals were kept isolated from the others. Afterward, challenged and non-challenged groups were housed in separate areas. Details on the vaccines and challenge inoculum composition and administration are indicated in the next subsection. This study aimed to include non-challenged, vaccinated groups, to enable the comparison of the immune responses elicited by the different immunization routes in non-challenged animals, something that was not possible in previous studies studying alternative vaccination routes (21, 33–35). This, and the logistical limitations, conditioned a small sample size of the groups.

The total duration of the experiment was 12 months. Blood sampling was performed monthly throughout the entire experiment, for the determination of the specific peripheral immune response. The injection sites of the IDV and SCV animals were also checked throughout the study. Fecal samples were taken 1, 5, and 10 months post-infection for *Map* fecal shedding quantification through qPCR, and at this time point, fecal consistency was checked. Additionally, fecal samples from the OV group were also collected at 3, 7, and 14 days post-vaccination (dpv). At the end of the study, all animals were euthanized, and tissue samples were taken for *Map* detection and lesion assessment (Figure 1).

Goat handling and blood sample collection were carried out in accordance with European Union legislation (Law 6/2013), concerning animals, their exploitation, transportation, experimentation, and sacrifice; R. D. 118/2021 for the protection of animals employed in research and teaching; Directive 2010/63/UE, related to the protection of animals used for scientific goals. All the procedures were approved by the corresponding animal welfare body (OEBA) and the Consejería de Agricultura y Ganadería de la Junta de Castilla y León (authorization code ULE-02-2021). All animals used in this study were handled in strict accordance with good clinical practices, and all efforts were made to minimize suffering.

### 2.2 Vaccines and challenge inoculum

Three different administration routes using the same *Map* strain (316F) with equivalent antigen quantity, but different adjuvants, were employed in this study. Both experimental vaccines contained 12.5 mg of antigen per dose to match Gudair^®^ antigen quantity as calculated by wet weight following guidelines for PTB vaccination trials (52). The experimental oral vaccine (OV) was composed of chemically inactivated *Map* and 5 mg of the adjuvant Quil A^®^ (InvivoGen, San Diego, CA, United States), diluted in 10 mL of physiological saline solution (B. Braun, Melsungen, Germany) and was administered orally using an automated syringe in two doses separated by 14 days. The efficacy of this vaccine and its effects on mucosal, trained, and innate immunity have been previously studied in the rabbit model (33). The experimental intradermal vaccine (IDV) was composed of chemically inactivated *Map* diluted in 0.4 mL of physiological saline solution and a single dose was administered, using a 25-G needle. The dose was inoculated in four previously shaved injection sites (0.1 mL per injection), two at each side of the dorsal thoracic area. The efficacy of this vaccine has been studied in the rabbit model, and its interference in the diagnosis of bTB through the skin test has been studied in guinea pigs (35). Finally, Gudair^®^, a commercial subcutaneous vaccine (SCV), composed of heat-inactivated *Map,* and a mineral oil-based adjuvant (containing Marcol 52, Montanide 80, and Montanide 103) was injected subcutaneously in a single 1 mL dose in the right ventral abdominal area, using an 18-G needle, as per manufacturer’s instructions.

The challenge inoculum, a low-passage type C field isolate of *Map* (strain 832) (58, 59), was grown on Middlebrook 7H9 broth enriched with 10% oleic acid–albumin–dextrose–catalase (OADC) and 2 mgL^−1^ Mycobactin J (7H9 OADC MJ) for 3 weeks at 37 ± 1°C. Then, cultures were harvested by centrifugation at 3,000 g for 10 min, and bacterial pellets were washed two times and resuspended in PBS. To disrupt bacterial clumps, the resultant suspension was passed up and down through a 27-G needle several times and vortexed. Bacterial concentration was estimated by optical density (OD) and colony-forming units (CFU) of 10-fold serial dilutions plated onto agar-solidified 7H9 OADC MJ. Finally, suspensions were adjusted to 2 × 10^10^ CFUs/ml, maintained at 4°C throughout the challenge period, and disrupted before oral inoculation as mentioned above. Specifically, each animal from the experimentally infected groups (INF) was orally inoculated with 8 × 10^9^ *Map* CFUs diluted in 10 mL of PBS, using an automatic syringe. The inoculation was performed four times separated by 3 days, as previously described by Fernández et al. (53), for a total dose of 3.2 × 10^10^ CFUs. Ten milliliters of PBS was administered orally to non-infected animals at the same time.

### 2.3 Sampling

Blood samples were monthly collected from the jugular vein into Vacutainer^®^ tubes (Becton Dickinson and Company, Erembodegem, Belgium) with lithium heparin and without anticoagulant. Then, heparinized samples were processed immediately for IFN-*γ* release test in response to PPDa and PPDb. Non-heparinized samples were processed for the study of the *Map-*specific antibody dynamics in the sera samples. Fecal samples were obtained per rectum using individual single-use polyethylene gloves and frozen at −80°C for quantification of *Map*-DNA through real-time quantitative polymerase chain reaction (qPCR).

At 11 mpv, all animals were humanely euthanized by deep sedation with xylazine (Xilagesic^®^, Calier, Barcelona, Spain) and subsequent intravenous injection of embutramide, mebezonium iodide, and tetracaine hydrochloride, (T61^®^, MSD Animal Health) followed by exsanguination. Then, a regulated, orderly, and complete necropsy was performed, and gross lesions were annotated. Immediately after gross examination of the viscera, a distal jejunal Peyer’s patch (DJPP), a draining jejunal lymph node (JLN), and the distal ileum (DI) were collected and frozen at either −20°C for *Map* culture or −80°C for quantification of *Map*-DNA through qPCR. In addition, tissue samples from the ileocecal valve, ileum [distal (DI), medial (MI), and proximal zones (PI)], jejunum [distal (DJ), medial (MJ), and proximal zones (PJ)], and Peyer’s patches [from the distal (DJPP), medial (MJPP), and proximal jejunum (PJPP)], together with ileocecal (ICLN), caudal mesenteric (CMLN), and jejunal (JLN)—lymph nodes, were taken, thoroughly washed in PBS, and taken into buffered formol saline fixative for histological examination.

### 2.4 Determination of the peripheral immune responses

To measure the *Map*-specific IgG in serum samples, the ID Screen^®^ Paratuberculosis Indirect (IDvet, Grabels, France) ELISA test was employed. Interpretation was performed following manufacturer’s instructions. Briefly, the optical density was measured spectrophotometrically at a wavelength of 450 nm (OD_450_) and the results were expressed as a ratio of the mean OD_450_ of each sample sera duplicates and the mean OD_450_ of the positive control sera duplicates from each plate (OD_450_ ratio).

For the determination of the specific cellular immune responses, within 3 h of blood collection, 1.5 mL aliquots of the heparinized blood samples were incubated in duplicate, in 24-well sterile plates, with either 100 μL of sterile phosphate-buffered saline (PBS), PPDa or PPDb (CZ Veterinaria, Porriño, Spain), at a final concentration of 20 μg/mL. After incubation (20 h at 37°C, 5% CO_2_), plates were centrifuged at 750 g for 15 min, and plasma was collected and stored at −20°C (60). Then, the assay for IFN-*γ* determination BOVIGAM^®^ TB Kit (Thermo Fisher Scientific, Waltham, MA, United States) was carried out in the plasma samples following the manufacturer’s instructions, interpreted as previously described (60), and results were expressed as a quotient (OD index), between the mean OD of the PPD-stimulated blood plasma and the mean OD of the plasma from the blood incubated with PBS.

Additionally, the standard interpretation of the BOVIGAM^™^ TB Kit was performed, to evaluate the possible interference of the different immunization strategies or the *Map* challenge with bTB diagnosis. According to the manufacturer’s instructions, PPDb-stimulated blood plasma with an OD value greater than 0.100 above that of both the PPDa and nil (PBS) antigen samples is indicative of the presence of *M. bovis* infection.

### 2.5 Histopathological examination and lesion classification

The formaldehyde-fixed tissue samples were conventionally processed for paraffin embedding and stained with hematoxylin–eosin (HE) for histopathological examination. Following the previously established classification for small animals, lesions consistent with PTB infection were classified as *focal*, when granulomas were restricted to the lymphoid tissue of PP; *multifocal*, when small granulomas were also located in the lamina propria; and *diffuse*, when the granulomas were widespread throughout the intestinal mucosa in some of the sections (19, 61). Additionally, three cross sections from the ileum (DI, MI, and PI), jejunum (DJ, MJ, and PJ), JPP (DJPP, MJPP, and PJPP), one longitudinal section from the ICV, and one section from each lymph node (ICLN, CMLN, and JLN) were selected. Then, the whole sections from ileal, jejunal, JPP, and ICV were analyzed, and the total number of granulomas per section was recorded. In the lymph nodes, a total of 5.46 mm^2^ of cortical area was analyzed per section (10 random 100× fields, Nikon^®^ Eclipse E600 microscope, coupled with a Nikon^®^ DS-Fi1 digital camera—Nikon, Tokyo, Japan).

### 2.6 Tissue and fecal detection and quantification of *Map*-DNA through qPCR

DNA was extracted from 50 mg of the homogenized tissue samples (DJPP, JLN, and DI) using the Maxwell^®^ RSC 16 Tissue DNA Purification Kit with the Maxwell^®^ RSC 16 Instrument (Promega, WI, United States), following the manufacturer’s instructions. Fecal DNA was extracted from 200 to 250 mg of homogenized fecal samples (three to four fecal pellets) using the Maxwell^®^ RSC Fecal Microbiome DNA Kit, with the Maxwell 16 Instrument (Promega, WI, United States), following the manufacturer’s instructions, including the optional bead-beating steps, which were performed using the Fisherbrand^™^ Bead Mill 24 (Thermo Fisher Scientific, Waltham, MA, United States). Thereupon, DNA was quantified using QuantiFluor^™^ ONEdsDNA System Kit (Promega, WI, United States) and Quantus^™^ Fluorometer (Promega, WI, United States). Extracted DNA was diluted to 50 ng · μL^−1^ for tissue samples and 20 ng · μL^−1^ for fecal samples and stored at −80°C until qPCR was performed, and a total of 100 ng of DNA were added to each qPCR reaction.

The primers employed for *Map* IS900 qPCR were: forward (MP10-1, [5′-ATGCGCCACGACTTGCAGCCT-3′]) and reverse (MP11-1, [5′-GGCACGGCTCTTGTTGTAGTCG-3′]) (62). The quantification was performed as described by Arteche-Villasol et al. (63), a 10-fold diluted standard curve was constructed using *Map* genomic DNA, obtained from 10^8^ CFUs of *Map* K10 strain, ranging from 10^−1^ to 10^−8^ ng of total DNA mixed with 20 ng of tissue DNA from a non-infected animal per reaction. Samples were analyzed in triplicate and considered as positive when the dissociation peak (Tm) was 89.1 ± 1°C and threshold cycles (Ct) were ≤ 37. The qPCR results were analyzed using 7,500 Software v2.0.6 (Applied Biosystems^™^). *Map*-DNA concentration (fg *Map*-DNA/g of tissue or feces) was calculated by interpolation of their Ct values with the standard curve, and results were expressed as the mean quantity of the triplicates.

### 2.7 *Map* detection through Ziehl–Neelsen and immunohistochemistry

The Ziehl–Neelsen (ZN) technique for acid-fast bacilli (AFB) detection and an immunohistochemical (IHC) staining against *Map* were used in formaldehyde-fixed paraffin-embedded tissue samples, for detecting *Map* bacilli and assessing its distribution. One DJPP, one JLN, and one DI section were employed for each technique. These tissues were chosen as they showed the most severe lesions (a mean of 53.6, 67.3, and 57.4 granulomas per section/area, respectively, in the challenged animals). The ZN staining was performed conventionally (19, 64), and sections were classified as positive or negative, based on the presence of AFB in the cytoplasm of macrophages.

The IHC staining was performed on 3-μm-thick tissue sections, placed onto poly-L-lysine-coated slides (SuperFrost Plus Adhesion slides, Thermo Fisher Scientific, Waltham, United States), as previously described (64). After deparaffinization and hydration, sections were washed two times using wash buffer (Agilent Technologies, Santa Clara, United States), for 5 min. Then, endogenous peroxidase was blocked by immersion of the sections into a 3% H_2_O_2_ in methanol for 30 min in darkness at room temperature. After washing two times, sections were incubated with an in-house rabbit anti-*Map* antibody (64), diluted 1:2000 in antibody diluent (Agilent Technologies, Santa Clara, United States), overnight, at 4°C, in a humidified chamber. After washing, sections were incubated for 40 min at room temperature with a secondary polyclonal antibody, horseradish peroxidase labeled polymer (Agilent Technologies, Santa Clara, USA), and after washing, antibody localization was determined using 3,3-diaminobenzidine (Agilent Technologies, Santa Clara, United States) as a chromogenic substrate for peroxidase. Sections were counterstained with Mayer’s hematoxylin for 10 s. Appropriate species- and isotype-matched immunoglobulins were used as negative controls. An ileal sample from a naturally *Map*-infected sheep with diffuse lesions was used as a positive control. IHC sections were classified as positive or negative, based on the presence of positively stained macrophages. Both ZN and IHC sections were observed under the Nikon^®^ Eclipse E600 light microscope, coupled with a Nikon^®^ DS-Fi1 digital camera (Nikon, Tokyo, Japan).

### 2.8 *Map* culture

DJPP, JLN, and DI samples from each animal were cultured for viable *Map* detection. Briefly, 1 g of each tissue was decontaminated with 19 mL of 0.8% hexadecylpyridinium chloride (Sigma-Aldrich, St. Louis, MO, United States) and homogenized in a stomacher blender (Masticator, IUL) for approximately 30 s. After 18 h of decontamination, 200 μL of the suspension was dispensed per tube containing Herrold’s egg yolk medium supplemented with sodium pyruvate and mycobactin J (MJ). Cultures were incubated at 37 ± 1°C, and growth was checked by examination under a stereoscopic microscope after 8, 12, 16, and 20 weeks post-inoculation. Cultures were considered positive if one or more characteristic *Map* colonies were observed in any tube. Colonies isolated were confirmed by a real-time multiplex PCR detecting IS900 and ISMap02 *Map* sequences (65).

### 2.9 Statistical analysis

The normality of the data was assessed using the Shapiro–Wilk test; all variables were non-normally distributed, so non-parametric tests were employed for the analysis. The antibody OD_450_ ratio and the avian and bovine indices were logarithmically transformed [log_2_(x + 1)]. Data collected at a single time point (PCR quantification of the tissue and fecal bacterial load, *Map* detection through culture, IHC results, and granuloma counts) were analyzed using Pearson’s chi-squared test of independence or Kruskal–Wallis test. Data collected at multiple time points (antibody OD_450_ ratio, the avian and bovine indices, and the *Map* fecal shedding quantification) were analyzed using the Friedman test. If statistically significant differences were detected, *post-hoc* tests were used to perform pairwise multiple comparisons: the Wilcoxon test (with Bonferroni–Holm correction for differences in total granuloma counts between groups) or the Dunn test. The Spearman rank test was performed to assess the correlation between the results of the different assays used for *Map* detection, and the results are included in Supplementary file S1. All statistical analyses were performed with the R software version 4.2.3 (66). The statistical packages psych (2.4.3), corrplot (0.92), rstatix (0.7.2), ggpubr (0.6.0.999), lubridate (1.9.2), forcats (1.0.0), stringr (1.5.0), dplyr (1.1.1), purrr (1.0.1), readr (2.1.4), tidyr (1.3.0), tibble (3.2.1), ggplot2 (3.4.2), and tidyverse (2.0.0) were used in the different statistical tests.

## 3 Results

### 3.1 Peripheral humoral and cell-mediated immune responses

**Figure 2:**
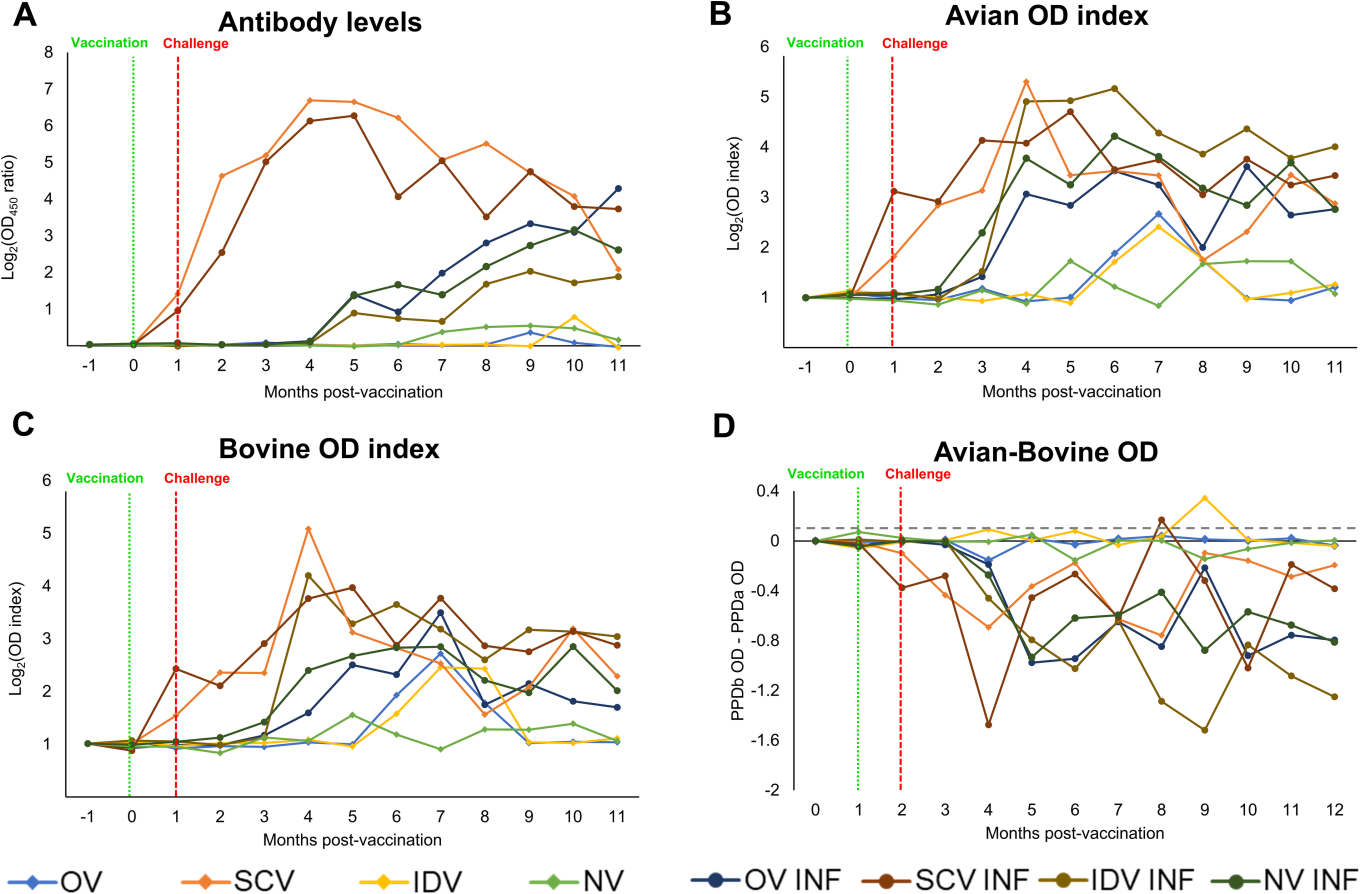
Kinetics of the peripheral immune response. **(A)** Dynamics of the specific anti-*Map* antibody response through the experiment, expressed as the mean Log_2_ (OD_450_ ratio) of each group. **(B)** Kinetics of the IFN-*γ* production by whole blood stimulated with PPDa. **(C)** Kinetics of the IFN-γ production by whole blood stimulated with PPDb, expressed as the mean avian and bovine Log_2_ (OD index) of each group. **(D)** bTB interference, calculated by subtracting the OD of the PPDa from the PPDb stimulated samples, results are expressed as a mean, and the gray horizontal dotted line represents the cutoff ratio of the BOVIGAM^™^ test (0.100). The experimental groups were as follows: OV (oral vaccine), OV-INF (oral vaccine, challenged), IDV (intradermal vaccine), IDV-INF (intradermal vaccine, challenged), SCV (subcutaneous vaccine), SCV-INF (subcutaneous vaccine, challenged), NV (non-vaccinated), and NV-INF (non-vaccinated, challenged). The vertically dotted red line represents the vaccine administration time-point, and the vertically dotted green line represents the challenge time-point. The results from the statistical analysis are shown in Supplementary Table S1.

Results from the indirect antibody ELISA are represented in Figure 2A, and its statistical analysis is provided in Supplementary Table S1. A rapid increase in the mean *Map*-specific antibody levels occurred after subcutaneous vaccination in the animals from the SCV and SCV-INF groups, with no apparent influence of the *Map* challenge in the latter group. A delayed, slower, and sustained increase took place in the remaining challenged groups (OV-INF, IDV-INF, and NV-INF), but no significant differences were detected between them. Regarding their non-challenged counterparts, animals in the OV and IDV groups did not show any increase in *Map*-specific antibody levels despite vaccination. In addition, animals in the NV group did not produce specific antibodies against *Map*.

The cellular immune responses (Figures 2B,C; Supplementary Table S1) were in line with the humoral response for most groups; in the SCV and SCV-INF groups, the avian and bovine index showed an early increase after vaccination. In contrast, in the other challenged groups (OV-INF, IDV-INF, and NV-INF), this increase in the cellular responses was slightly delayed, starting 2 to 3 months after the infectious challenge, and was sustained up to the end of the experiment. However, the OV and IDV groups behaved differently, with only subtle and time-limited cellular responses initiating at a late time point of approximately 5–6 months after vaccination. The mentioned increases in the avian and bovine OD index values were significant (*p* < 0.05), with respect to the basal levels, at some point in all groups except for the NV group. This increase took place sooner in the challenged groups, and overall, in all groups, higher mean values were reached for the avian index. Throughout the experiment, at several time points, all the challenged groups, except for the SCV-INF, showed significantly higher avian and bovine indexes (*p* < 0.05) than their respective only challenged counterparts.

Following the standard interpretation of the BOVIGAM™ TB Kit, several animals would have been considered positive for bTB (PPDb OD–PBS OD ≥ 0.1 and PPDb OD–PPDa OD ≥ 0.1) at some point during the experiment. Specifically, one from the SCV group (animal 52 at 2 MPI and 4 MPI), two from the IDV group (animals 31 and 32 at 7 MPI), all three animals from the SCV-INF group (animal 61 at 4 MPI, 62 at 6 and 7 MPI, and 63 at 6 MPI), and one animal from the NV-INF group (animal 81 at 8 MPI). The quantitative results of the PPDb OD-PPDa OD subtraction are represented in Figure 2D.

### 3.2 Clinical and pathological findings

In the SCV group, vaccine injection site nodules, which were already present since the first few weeks after vaccination, persisted at the time of necropsies. However, animals from the IDV group did not develop any noticeable lesions at the injection site during the experiment. Fecal consistency remained unaffected in all animals, exhibiting firm, well-formed pellets at all the selected time points (1, 5, and 10 mpi).

Regarding gross lesions, none of the non-challenged animals showed any lesion during necropsies, whereas gross lesions compatible with PTB were observed in the ileum, jejunum, and mesenteric lymph nodes of some of the challenged animals except for the SCV-INF group. The most affected group was the non-vaccinated (three of seven animals), whereas only one of three animals showed gross lesions in the remaining groups (OV-INF and IDV-INF) (Table 1). The main gross lesions observed were a diffuse thickening of the ileal and/or jejunal mucosa (Supplementary file S2A) and cortical enlargement of the mesenteric lymph nodes (Supplementary file S2B). In some of the challenged goats, in addition to the diffuse thickening, congestion was observed in some of the Peyer’s patches (Supplementary file S2D) and one of the animals from the OV-INF group presented multiple foci of caseous necrosis, located in the Peyer’s patches (Supplementary file S2C).

**Table 1:** Lesion assessment in infected groups.

Group	Animal	Gross lesions	Histopathological classification	Granuloma count								Mean no. of granulomas^b^
				ICV	ICLN	I (Mean^a^)	CMLN	J (Mean^a^)	JPP (Mean^a^)	JLN	Total	
OV-INF	21	No	Focal	0	0	0	0	0	0.7	0	2	31.69** ± 8.86
	22	No	Multifocal	2	0	2.3	1	9.7	4	40	91	
	23	Yes	Diffuse	5	48	140	115	104.7	19.7	182	1,143	
IDV-INF	41	No	Diffuse	4	3	1	0	20	49.3	57	242	42.79 ± 9.89
	42	Yes	Diffuse	157	128	23	186	150	103.7	180	1,321	
	43	No	Multifocal	7	2	2	50	2.7	5	12	106	
SCV-INF	61	No	Diffuse	15	2	58.7	3	20.3	16	12	303	18.23* ± 4.07
	62	No	Multifocal	2	4	9.7	10	4.7	4.7	1	61	
	63	No	Diffuse	39	0	22.7	0	16.3	28.3	0	317	
NV-INF	81	No	Multifocal	8	9	3.3	11	18	28.3	2	186	40 ± 5.92
	82	Yes	Diffuse	13	17	8	90	21.7	94.7	195	692	
	83	Yes	Diffuse	46	33	114.3	107	59.3	124	53	1,204	
	84	No	Diffuse	44	25	44.7	14	7.3	24	19	405	
	85	No	Diffuse	5	6	14.3	7	49.0	81.7	139	737	
	86	No	Multifocal	0	7	4	14	1.3	2	13	87	
	87	Yes	Diffuse	7	15	11.7	2	26.3	55.7	14	329	

Presence of gross lesions consistent with paratuberculosis, histopathological lesion classification, and results of the granuloma counts. I, J, and JPP counts are expressed as the mean number of granulomas from three different sections (proximal, medial, and distal). ICV (ileocecal valve), ICLN (ileocecal lymph node), I (ileum), CMLN (caudal mesenteric lymph node), J (jejunum), JPP (jejunum Peyer’s Patch), and JLN (jejunal lymph node). Significant differences with the NV-INF group are indicated groups are indicated with an asterisk where * and ** denote *p* < 0.05 and *p* < 0.01, respectively.

**Figure 3:**
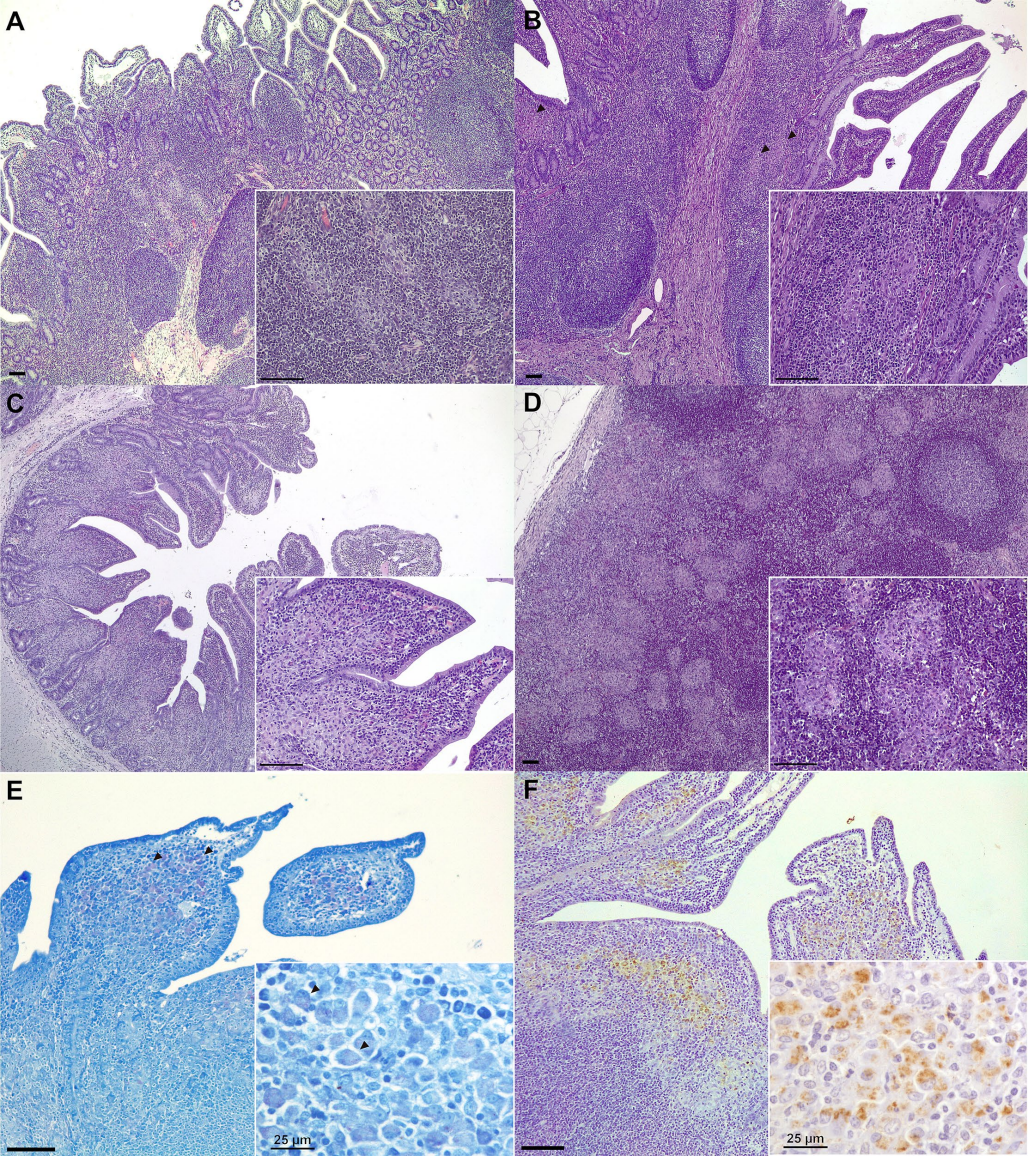
Representative microscopic lesions and *Map* detection in tissue samples. **(A)** Focal lesion: multiple well-demarcated granulomas located in the interfollicular area of the Peyer’s patches (medial ileum, Goat 64, SCV-INF group); **(B)** Multifocal lesion: granulomas (arrowheads) can be seen in both the lymphoid tissue and lamina propria (medial jejunum Peyer’s patch, Goat 22, OV-INF group); **(C)** Diffuse lesion: the lamina propria is a diffuse granulomatous infiltrate affects the lamina propria (medial jejunum, Goat 42, IDV-INF group). **(D)** Multifocal to coalescing granulomatous foci present in the cortex of a jejunal lymph node (goat 83, NV-INF group). Representative images of *Map*-positive samples as detected through: **(E)** Ziehl–Neelsen staining. Some acid-fast staining can be observed in some of the macrophages present in the apex of the villi (arrowheads) (distal jejunum Peyer’s patch, Goat 82, NV-INF group). **(F)** Immunohistochemistry. Macrophages positive to *Map* immunostaining are observed in the diffuse granulomatous infiltrate affecting the lamina propria over the lymphoid tissue of a distal jejunum Peyer’s patch (distal jejunum Peyer’s patch, Goat 82, NV-INF group). Unless indicated otherwise, scale bars equal to 100 μm.

Microscopic lesion classification and granuloma counts for each challenged animal and tissue are also indicated in Table 1. All challenged animals developed microscopic granulomatous lesions despite vaccination; however, a wide range of lesion severity was observed (Figures 3A–D), both between individuals and between different tissues in the same individuals (Table 1). The mean number of granulomas was significantly lower in the OV-INF (*p* < 0.01) and SCV-INF (*p* < 0.05) groups with respect to the NV-INF. Additionally, the number of granulomas present in the ICLN and JLN lymph nodes from the animals in the SCV-INF group was significantly lower (*p* < 0.05) than that found in the animals from the NV-INF group. Overall, microscopic lesion severity was high; of the 16 challenged animals, 10 developed diffuse lesions and 5 developed multifocal lesions, and the tissues with the highest number of granulomas were DJPP, JLN, and DI, with a mean of, 67.3, 57.4, and 53.6 granulomas per section/area, respectively, in the challenged animals.

### 3.3 *Map* fecal shedding

After oral vaccination, *Map-*DNA pass-through was detected in at least one sampling during the first 14 dpv in five out of six vaccinated animals. By 3, 7, and 14 dpv, *Map-*DNA was detected in four of six, one of six, and two of six of the orally vaccinated animals, respectively, in each time point. The mean DNA quantified was low in all samplings: 0.97 ± 0.34, 0.22 ± 0.22, and 0.57 ± 0.37 fg of *Map-*DNA/g feces, respectively.

After the challenge, the evolution of *Map* fecal shedding can be seen in Table 2. Before infection, all fecal samples from non-challenged animals were negative throughout the entire experiment, except for the OV group as mentioned above. After infection, in challenged animals, inter- and intraindividual variability in *Map* fecal shedding was very high, and no significant differences between challenged groups were detected. The number of animals shedding and the mean *Map-*DNA quantity shed progressively increased throughout the experiment; this increase was only significant in the NV-INF group (*p* < 0.05). Specifically, at 1 month post-infection (mpi), two of three animals from the IDV-INF group, and four of seven from the NV-INF group were positive and the remaining were negative. By 5 mpi, two of three animals from the OV-INF group, one of three animals from the IDV-INF group, two of three animals from the SCV-INF group, and four of seven animals from the NV-INF group were positive by qPCR. By the end of the experiment (10 mpi), all fecal samples from challenged animals, except for one animal from the NV-INF group and another from the SCV-INF group, were positive to *Map.*

**Table 2:** *Map* detection and quantification in feces and tissues in challenged animals.

Group	Animal	Fecal shedding (fg *Map*-DNA/g feces)				Tissue bacterial load (fg *Map*-DNA/g tissue)					IHC and ZN*			Culture		
		1 mpi	5 mpi	10 mpi	Group mean (by 10 mpi)	DJPP	JLN	DI	Mean	Group mean	DJPP	JLN	DI	DJPP	JLN	DI
OV-INF	21	0.00	0.00	1.15	1632.9 ± 1617.78	1.56	0.00	0.33	0.63	1362.1 ± 1270.46	−	−	−	−	−	−
	22	0.00	39.01	29.95		325.11	224.14	0.00	183.08		+	−	−	−	+	+
	23	0.00	731.79	4867.71		1964.79	438.28	9304.25	3902.44		+	−	+*	+	+	+
IDV-INF	41	0.22	0.00	66.33	757.85 ± 722.16	771.38	9.56	0.00	260.31	131.89 ± 75.08	+	−	+	−	+	+
	42	0.04	813.95	2200.43		392.30	3.98	8.85	135.05		+	−	+	+	−	+
	43	0.00	0.00	6.80		0.00	0.90	0.00	0.30		−	−	−	−	−	−
SCV-INF	61	0.00	0.00	1.46	17.78 ± 17.06	0.50	0.05	1.57	0.70	26.31 ± 24.27	−	−	+	−	−	−
	62	0.00	1.97	0.00		0.62	8.01	1.61	3.41		−	−	−	−	−	−
	63	0.00	2.44	51.87		105.58	17.86	101.07	74.83		+	−	−	+	−	+
NV-INF	81	0.64	0.00	5.79	38.97 ± 15.52	2.86	0.00	0.65	1.17	207.91 ± 128.1	−	−	−	−	−	−
	82	0.00	0.94	83.41		246.72	9.66	0.00	85.46		+*	−	−	−	−	+
	83	0.27	0.00	80.06		326.17	14.01	23.33	121.17		+	+	+	+	−	+
	84	1.67	17.90	11.29		23.20	1.57	2.72	9.16		−	−	+	−	−	+
	85	0.06	5.33	84.66		889.58	6.41	3.96	299.99		+*	−	+	−	−	−
	86	0.00	0.00	0.00		0.00	1.56	0.00	0.52		−	−	−	−	−	−
	87	0.00	134.14	7.60		2793.89	19.86	0.00	937.92		+	−	−	−	−	−

Quantification of *Map* fecal shedding by 1, 5, and 10 mpi (months post-infection) and tissue bacterial load at the end of the experiment through qPCR, expressed as fg *Map*-DNA/g tissue. *Map* detection in tissues through the ZN (Ziehl–Neelsen) staining, IHC, and bacteriological culture. *Positive samples to ZN staining. DJPP, distal jejunal Peyer’s patch; JLN, jejunal lymph node; DI, distal ileum.

### 3.4 *Map* detection in tissues

The bacterial load per tissue, as assessed through qPCR as well as ZN, IHC, and *Map*-culture results can be seen in Table 2. The tissue bacterial load results align with the fecal shedding data, as *Map* was detected via qPCR in at least one tissue sample from all challenged animals (Table 2). In addition, in accordance with the microscopic lesions observed, the samples with a higher *Map*-DNA load in most animals (11/16) were the DJPP, and the SCV group showed the lowest mean *Map* load. However, the *Map* load varied greatly both between samples and animals, and no significant differences were observed in tissue distribution or between groups.

In this line, *Map* was detected through bacteriological culture, in at least one tissue, in one of three animals from the SCV-INF group in two of three animals from the OV-INF and IDV-INF groups and in three of seven animals from the NV-INF group (results are indicated in Table 2), but no significant relation between culture detection of *Map* and the vaccine employed in challenged animals was detected by the chi-square test (*p >* 0.05).

Representative examples of ZN and *Map* IHC positive staining can be seen in Figures 3E,F, respectively, and the results for the individual animals and tissues are collected in Table 2. AFB was only observed in a small number of macrophages, in extensive areas of granulomatous infiltrate in samples from one of three and two of seven animals from the OV-INF and NV-INF groups, respectively. *Map* was detected through IHC mostly in DI and DJPP sections; positively stained macrophages were mainly present in the diffuse granulomatous infiltrates and large granulomas. Two out of three animals from the OV-INF and IDV-INF groups, 1/3 from the SCV-INF group, and 6/7 from the NV-INF group were positive to *Map* IHC. No significant relation between the immunohistochemical detection of *Map* and the vaccine employed in challenged animals was detected by the chi-square test (*p >* 0.05).

The detection of *Map* in feces and tissues, through the different techniques employed, showed strong correlations in most cases (Supplementary Figure S1). An interesting exception was the results from the JLN samples, as most of them were negative to IHC and culture.

## 4 Discussion

Alternative routes of vaccination like the oral and intradermal are new strategies that could aid to circumvent some of the drawbacks of the currently available subcutaneous vaccines against paratuberculosis. Few studies have been made on ruminants on vaccines employing the oral route and most of them used live-attenuated, non-adjuvanted vaccines (21, 34, 51). Furthermore, the intradermal route of vaccination against paratuberculosis remains unexplored in ruminants. Thus, the present study has compared the effect on the specific peripheral immune responses and the protective efficacy of two experimental PTB vaccines, administered through the oral and intradermal routes, with that of a commercial SCV. As expected, the SCV vaccine induced strong peripheral immune responses which remained unaffected by the subsequent *Map* challenge. Conversely, mild, delayed, and short-spanned peripheral cellular responses were induced by the OV and IDV, suggesting its potential of not interfering with bTB diagnosis. However, although none of the three vaccines conferred complete protection against the experimental *Map* challenge, the SCV (Gudair^®^) was the most effective at reducing bacterial load, fecal shedding, and lesion severity, whereas the OV reduced lesion severity, and the IDV reduced tissue bacterial load, when compared to the NV-INF animals.

The efficacy of these immunization strategies has been previously tested in rabbits (33, 35), and its interference with bTB diagnosis has been assessed in guinea pigs (35). In those experiments, the IDV induced a higher bacterial clearance and lower bTB diagnosis interference than the SCV control. Additionally, the OV-induced mucosal immunity (significantly increasing TNF, IL-1β, IL-10, IL-12B, and IL-23A expression in the GALT) (33) and a comparable OV, albeit without adjuvant, induced similar clearance to that of the control vaccine while not interfering with bTB diagnosis (35). However, it is important to emphasize that experimental results obtained in laboratory animal models of paratuberculosis vaccination do not always correlate with those observed in ruminants (21). Because of this, studies in the relevant host are necessary to assess vaccine efficacy, and because of its pathogenesis, long-term studies are also necessary to study *Map* infection progression. Cattle require significantly higher upkeep and larger facilities than small ruminants, whereas goats are more susceptible to developing clinical PTB than sheep, particularly to type C *Map* strains (54, 55, 67, 68). Additionally, goats are also more susceptible to bTB infection (69, 70). Therefore, the caprine model was considered the most suitable for studying the efficacy of PTB vaccination and its interference with bTB diagnosis. Nevertheless, PTB is difficult to model, starting from the challenge inoculum, as culture passages significantly decrease *Map* virulence (71, 72), and therefore, a common problem faced by experimental infections is the lack of infectivity and virulence of cultured strains (73). In addition, the short time periods used in most experimental settings, of a few months versus the several years that natural infections would require to produce severe lesions and reach the clinical disease stage (68, 74), further complicate the interpretation of the challenge outcome. To solve these challenges, in the present study, we have used a low-passage, type C field *Map* isolate, at a relatively high dose for the experimental infection and a study duration of 10 months post-challenge. This has resulted in a high infection rate of the challenged animals; however, tissue bacterial load, *Map* shedding, and lesion severity were highly variable between individuals, which would be in concordance with that observed in natural and experimental infections (12, 75, 76).

The peripheral immune response elicited by PTB infection is characterized by an initial strong cell-mediated immune response, followed by a late, steady increase in the humoral immune response (77). This switch is generally thought to signify a breakdown in disease control (78) and can be seen in the evolution of the response developed by the animals from the infected groups. This pattern of response was followed by the OV-INF and IDV-INF animals but not by their non-challenged counterparts, which suggests that these peripheral responses could be elicited by infection and were not affected by vaccination. The lack of induction of humoral responses could be expected, particularly in oral vaccines, as they primarily stimulate local (intestinal) immunity (79). In this sense, the non-challenged OV- and IDV-vaccinated goats did not produce significant levels of specific IgG at any point in the 10 mpv period. Similarly, in a previous experiment carried out by Hines et al. (34), using live-attenuated oral vaccines in goats, antibody production started 5 months after the *Map* challenge, which suggests that it was not elicited by vaccination but by the subsequent challenge. In line with this, Arrazuria et al. (35) also observed an absence of humoral responses during 160 dpv, in orally or intradermally vaccinated rabbits.

This humoral immunity has not been traditionally considered necessary for protection against *Map* infection, although some recent studies point out in the opposite direction (78). On the other hand, the cell-mediated response, and particularly Th1, is widely considered critical in the effective response against mycobacterial infections (80). In our experiment, in the OV and IDV groups, a peak in the peripheral cell-mediated immune responses against avian and bovine PPDs was observed between 6 and 8 mpv. This peak was short-lived, of low intensity, and occurred late with respect to the response induced by subcutaneous vaccination or infection. In this sense when applying the standard interpretation of the BOVIGAM^™^ test, by 7 MPI, two of the IDV-vaccinated animals would be considered positive to bTB, but no OV, OV-INF, or IDV-INF animal was positive throughout the experiment. Few studies can serve as a comparison; in the mentioned study carried out by Arrazuria et al. (35), for example, the cell-mediated response was only measured through skin testing in the guinea pig model, and in this case, the animals vaccinated through the oral route did not show skin reaction against both PPDa and PPDb, but the intradermal vaccine induced a significant skin reaction against both PPD, although it was lower than that of Silirum®. Furthermore, a study carried out in goats testing the same oral vaccine showed the absence of interference with PPDa and PPDb in the skin test 48 days post-vaccination (81). In accordance with the results presented, oral, whole-cell heat-inactivated bTB vaccines did not induce specific antibody production or cell-mediated immune responses before the *M. bovis* challenge, in goats (39), cattle (46), red deer (82), or boar (83). As previously stated, no intradermal PTB vaccines have been tested in ruminants; however, the subtle peripheral responses observed contrast with the strong cell-mediated responses induced by several intradermal vaccines against human tuberculosis. Nevertheless, without considering species differences, the formulation and/or posology of these vaccines differ substantially from the one tested in this study, with most studies employing attenuated or vectored vaccines (43, 45) or multiple doses of an inactivated vaccine (84).

The peripheral responses induced by the control SCV were substantially different from those induced by the experimental vaccines; strong humoral and cell-mediated immune responses were detectable early after vaccination in the subcutaneously vaccinated animals, reaching their peak by 3–5 mpv. This is the typical pattern of response induced by Gudair^®^ (85) and other inactivated whole-cell, oil-adjuvanted vaccines like Silirum^®^ (86) and Mycopar^®^ (87). In addition, in contrast with the experimental vaccines, the infectious challenge did not significantly affect the kinetics of the peripheral immune response in the animals of the SCV-INF group. Regarding the interference of this vaccine with bTB diagnosis, as per BOVIGAM standard interpretation, only one SCV animal was positive by 4 MPI, but the three SCV-INF animals were positive at least once during the experiment. This vaccine (88) and PTB exposure or infection (89) are known to interfere with bTB diagnosis. In field conditions, both factors are present, as PTB-vaccinated herds are those where *Map* is circulating, so vaccinated animals are likely exposed to *Map* and therefore prone to yielding false positives to bTB diagnostic tests. Overall, regarding the peripheral immune responses, it should be stated that they do not always perfectly align with the mucosal responses (90, 91). In this sense, our general knowledge of the local immunity elicited by PTB vaccination, particularly through the oral route, is still very limited.

Herein, the intestinal gross lesions observed in some of the animals are compatible with typical PTB lesions, including the caseous necrotic areas and lymphangiectasia, which constitute a specific finding of small ruminants, usually found in naturally infected animals (19, 92, 93). These findings suggest the effective establishment of *Map* infection in some of the challenged animals and the usefulness of the caprine model for the study of PTB. Microscopic lesion classification revealed a wide range of severity, indicating varied individual and tissue-specific responses to infection. Despite this high variability, the overall progressive increase in shedding quantity, and the detection of *Map* through different techniques and in multiple tissues, further suggest the successful establishment and persistence of the infection in animals from all groups. However, the significantly lower granuloma counts in the OV-INF and SCV-INF groups, when compared to the NV-INF group, indicate potential differences in disease progression or immune control among these groups, in spite of the differences observed in the peripheral immune responses between both. This immune control is further supported in the SCV-INF group, where the lower number of granulomas present in the mesenteric lymph nodes (JLN, CMLN, and ICLN), with respect to the NV-INF group suggests a more effective containment of *Map* spread in the intestine. In this sense, interestingly, the low number of JLN samples found positive through *Map* IHC and culture in all groups, suggests that *Map* integrity was low in this tissue and that a large proportion of the *Map*-DNA detected in this tissue may pertain to degraded bacteria.

In general terms, the diffuse lesions observed, which are considered the pathological form of patent PTB disease (94), together with the continuous increase in fecal shedding and/or high tissue bacterial load, suggest that the disease is progressing in animals 22, 23, 41, 42, 63, 82, 83, 84, 85, and 87. This would mean that none of the vaccines was able to completely protect animals from PTB infection and lesion development despite differences in bacterial load, shedding, and lesion severity. Similarly, previous studies have demonstrated partial protection against *Map* or *M. bovis* infection conferred by OV and IDV, but when compared with parenterally administered vaccines, they were often outperformed. For example, in rabbits challenged with a different, less virulent strain, Silirum^®^ reduced PTB lesion severity more than IDV and OV (35). In goats, two of five live-attenuated OV tested slightly decreased lesion severity, but none of them had a significant effect on *Map* fecal shedding when compared to non-vaccinated controls, and all were outperformed by Silirum^®^ in both parameters (34). In Eurasian boar, both an inactivated *M. bovis* vaccine and live BCG, administered orally, reduced lesion severity but both were outperformed by a vaccine administered parenterally (83). In red deer, these vaccines were not compared with a parenterally administered one, but they only conferred partial lesion reduction (95). As mentioned earlier, most of these IDV and OV vaccines induced poor peripheral immune responses. While reducing the diagnostic interferences caused by the peripheral responses induced by subcutaneous vaccines would be beneficial, those findings suggest that eliminating the peripheral responses elicited by vaccination might compromise protection. Further studies are needed to better understand these mechanisms.

Histopathological and tissue bacterial load assessments are however constrained, providing insights on *Map* infection at a single time point. This, coupled with the substantial variability in *Map* distribution across the intestinal tract, and the intricate dynamics of PTB pathogenesis, presents a challenge to understanding the ongoing processes in the infected individuals. In addition, the differences in qPCR, IHC, ZN, and culture results emphasize the complexity of assessing *Map* presence and distribution, and the need for multiple diagnostic approaches, being the qPCR the most sensitive technique for direct *Map* detection. However, it should be noted that this method is not able to assess the viability of *Map* in feces, which would indicate whether the vaccine is reducing the risk of transmission by reducing viable *Map*. In addition, in the light of the results, to better analyze the effect of the alternative strategies tested in this study, a higher sample size would have been needed due to the high inter- and intraindividual variability observed, not only in lesion severity but also in the fecal shedding patterns and tissue bacterial load. This finding was expected, the variability observed in lesion severity and tissue bacterial load is characteristic of *Map* infection, even in non-vaccinated, naturally infected animals (19, 96). Moreover, differences between low and high shedders are often several orders of magnitude apart (68), because of this, the results of all the individual animals have been presented, instead of plotting the means. In this sense, for example, animal 23 (OV-INF) exhibited exceptionally high levels of *Map*-DNA in the studied tissues, surpassing all other animals. In this line, it tested positive for *Map* culture in all tissues, demonstrated the highest levels of *Map*-DNA fecal shedding, and showed elevated granuloma counts in all tissues. Though this could be an incidental result, a recent study in European badgers (*Meles meles*), found that two out of eight of the animals, vaccinated with an inactivated OV against *M. bovis,* responded in a clearly divergent manner to this vaccination strategy, showing increased lesion severity and bacterial load. The authors proposed several hypotheses like tolerization induced by the oral vaccine or T-cell exhaustion (97).

In this study, to compare the efficacy of each immunization route, the same amount of antigen was administered in the three tested vaccines. However, it needs to be pointed out that a well-designed intradermal vaccine should be effective using lower amounts of antigen than a subcutaneously administered one (47), whereas oral vaccines require higher antigen doses or multiple administrations, as used for human oral vaccines, to achieve the same potency as injected vaccines, due to degradation in the digestive system (79). Thus, the results here presented should be different by adjusting the amount of antigen according to the route of immunization. In this sense, as mentioned before, inactivated OV vaccines have been tested with some success in a rabbit model (33, 35), leaving aside the differences between rabbits and ruminant’s immune systems, rabbits are monogastric and show considerably shorter retention times of ingesta and cecotrophy (98, 99). In contrast, the extended retention times in the upper digestive tract in ruminants mean that a significantly greater quantity of antigen can be potentially degraded in the forestomachs before reaching the intestinal gut-associated lymphoid tissue (GALT), the primary site for antigen uptake, processing, and initiation of immune responses in the intestine (79). To increase this OV effectiveness, an adequate delivery system, targeted for release in the small intestine, could be used to protect both the antigen and the adjuvant from degradation in the gastrointestinal tract (100). Additionally, a higher dose should compensate for the possible degradation and the differences in live weight between rabbits and ruminants. Regarding the OV vaccine, fecal shedding of *Map-*DNA, by most of the orally vaccinated animals, was detected throughout the first 2 weeks after vaccine administration. Moreover, even though the shed quantity was low, it would be of concern in a live-attenuated vaccine and highlights one of the main potential limitations of the oral administration of this type of vaccine. Regarding IDV, multiple doses could be administered to improve its immunogenicity, given that the amount of antigen inoculated at each injection site cannot be increased. However, it would also incur increased costs. Alternatively, the addition of an adjuvant could increase the IDV potency (101–103). Up to the present, most currently approved adjuvants have been considered as not suitable for intradermal delivery due to the high risk of local reactions (101). However, recently, several adjuvants, some of them previously approved for subcutaneous or intramuscular delivery, have been proven to be safe for use in intradermal vaccines (102, 103), including Quil A^®^, and its purified fraction QS-21 (104). Quil A^®^ has also been tested in a live-attenuated subcutaneous vaccine against PTB, and it induced a strong IFN-*γ* response in the goat model without antibody production, and showed significant protective efficacy, reducing histopathological lesions, challenging strain tissue colonization and eliminating fecal shedding (105). The strong IFN-γ response induction, along with the reduction in fecal shedding, was confirmed in a bovine model. However, in this model, the challenge strain employed did not establish itself in the host tissues in any of the study groups (106). Further studies, testing an inactivated subcutaneous Quil A^®^-adjuvanted vaccine could provide valuable information.

To conclude, new immunization strategies against PTB have been tested in a caprine model confirming that the oral and intradermal immunization routes barely influence the *Map-*specific peripheral immune responses and therefore induce a low and predictable interference with the bTB IGRA testing. Even though the OV reduced lesion severity, and the IDV reduced tissue bacterial load, both experimental prototypes were outperformed by the SCV; these results and those of previous studies suggest that achieving vaccine protection against PTB in ruminants without inducing peripheral responses is likely complicated. Alternative PPDs, molecularly defined antigens, as well as DIVA vaccines and diagnostic techniques, may provide a solution to reduce diagnostic interferences (5, 107); however, implementing these options would require changes in the current legislation. Nevertheless, the peripheral immune response parameters induced by vaccination and challenge, in the present study, were not predictive of the infection outcome or lesion severity in the challenged animals. Thus, further investigation into the significance of the peripheral responses induced by these alternative vaccines in protection against PTB, and its effect in the local immunity, would be of interest, especially given the mentioned lack of correlation between the peripheral and mucosal responses induced by parenteral vaccines (90, 91). Our general knowledge on the local immunity elicited by PTB vaccination, particularly through the oral route, is still very limited. It would be of interest to analyze both the local cellular (e.g., cytokine expression and immune cell subpopulations) and humoral responses (e.g., IgA production and B-cell subpopulations), established after vaccination and infection, using larger sample sizes and longer terms, in further studies. This could aid in the development of alternative vaccination strategies that offer protection without the compromises of currently available vaccines.

## Data Availability

The original contributions presented in the study are included in the article/Supplementary material, further inquiries can be directed to the corresponding author.
