# Correction: Evaluation of alternative vaccination routes against paratuberculosis in goats

**DOI:** 10.3389/fvets.2026.1850598

**Published:** 2026-04-29

**Authors:** Miguel Criado, Marta Silva, Noive Arteche-Villasol, David Zapico, Natalia Elguezabal, Elena Molina, José Espinosa, María del Carmen Ferreras, Julio Benavides, Valentín Pérez, Daniel Gutiérrez-Expósito

**Affiliations:** 1 Departamento de Sanidad Animal, Instituto de Ganadería de Montaña (IGM) CSIC-ULE, León, Spain; 2 Departamento de Sanidad Animal, Facultad de Veterinaria, Universidad de León, Campus de Vegazana, León, Spain; 3 Departamento de Sanidad Animal, NEIKER-BRTA, Instituto Vasco de Investigación y Desarrollo Agrario, Derio, Spain

**Keywords:** paratuberculosis, oral vaccine, intradermal vaccine, immunization strategies, goat

In the published article there was a mistake in Table 2. The standard error of the tissue bacterial load for the IDV-INF and SC-INF groups, as well as the mean and standard error of both fecal shedding and tissue bacterial load for the NV-INF group, were miscalculated. The corrected table and its caption appear below.

**Table 2:** Map detection and quantification in feces and tissues in challenged animals.

Group	Animal	Fecal shedding (fg *Map* DNA/g feces)				Tissue bacterial load (fg *Map* DNA/g tissue)					IHC and ZN^*^			Culture		
		1 mpi	5 mpi	10 mpi	Group mean (by 10 mpi)	DJPP	JLN	DI	Mean	Group mean	DJPP	JLN	DI	DJPP	JLN	DI
OV-INF	21	0.00	0.00	1.15	1632.9 ± 1617.78	1.56	0.00	0.33	0.63	1362.1 ± 1270.46	–	–	–	–	–	–
	22	0.00	39.01	29.95		325.11	224.14	0.00	183.08		+	–	–	–	+	+
	23	0.00	731.79	4867.71		1964.79	438.28	9304.25	3902.44		+	–	+^*^	+	+	+
IDV-INF	41	0.22	0.00	66.33	757.85 ± 722.16	771.38	9.56	0.00	260.31	131.89 ±**75.08**	+	–	+	–	+	+
	42	0.04	813.95	2200.43		392.30	3.98	8.85	135.05		+	–	+	+	–	+
	43	0.00	0.00	6.80		0.00	0.90	0.00	0.30		–	–	–	–	–	–
SCV-INF	61	0.00	0.00	1.46	17.78 ± 17.06	0.50	0.05	1.57	0.70	26.31 ±**24.27**	–	–	+	–	–	–
	62	0.00	1.97	0.00		0.62	8.01	1.61	3.41		–	–	-	–	–	–
	63	0.00	2.44	51.87		105.58	17.86	101.07	74.83		–	–	–	+	–	+
NV-INF	81	0.64	0.00	5.79	**38.97** **±15.52**	2.86	0.00	0.65	1.17	**207.91** **±128.1**	–	–	–	–	–	-
	82	0.00	0.94	83.41		246.72	9.66	0.00	85.46		+^*^	–	–	–	–	+
	83	0.27	0.00	80.06		326.17	14.01	23.33	121.17		+	+	+	+	–	+
	84	1.67	17.90	11.29		23.20	1.57	2.72	9.16		–	–	–	–	–	+
	85	0.06	5.33	84.66		889.58	6.41	3.96	299.99		+^*^	–	+	–	–	–
	86	0.00	0.00	0.00		0.00	1.56	0.00	0.52		–	–	–	–	–	–
	87	0.00	134.14	7.60		2793.89	19.86	0.00	937.92		+	–	–	–	–	–

Quantification of Map fecal shedding by 1, 5, and 10 mpi (months post-infection) and tissue bacterial load at the end of the experiment through qPCR, expressed as fg Map-DNA/g tissue. Map detection in tissues through the ZN (Ziehl-Neelsen) staining, IHC, and bacteriological culture. ^*^Positive samples to ZN staining. DJPP (distal jejunal Peyer's patch), JLN (jejunal lymph node), and DI (distal ileum). Corrections have been highlighted in bold.

A correction has been made to the **Abstract**. Sentence previously stated:

“Although the OV reduced lesion severity, neither this vaccine nor the IDV prototype was able to reduce fecal shedding or tissue bacterial load”.

The correct sentence appears below:

“Although both experimental prototypes were not able to reduce fecal shedding, the OV reduced lesion severity, whereas the IDV prototype reduced the tissue bacterial load.”

A correction has been made to the **Discussion**, paragraph one. Sentence previously stated:

“… the SCV (Gudair^®^) was the most effective at reducing bacterial load, fecal shedding and lesion severity; whereas the OV reduced lesion severity when compared to the NV-INF animals, and the IDV failed to reduce any of these protection parameters.”

The correct sentence appears below:

“… the SCV (Gudair^®^) was the most effective at reducing bacterial load, fecal shedding, and lesion severity, whereas the OV reduced lesion severity, and the IDV reduced tissue bacterial load, when compared to the NV-INF animals.”

A correction has been made to the **Discussion**, paragraph ten. Sentence previously stated:

“Even though the OV reduced lesion severity, the IDV did not show any evidence of protective efficacy, and both were outperformed by the SCV....”

The correct sentence appears below:

“Even though the OV reduced lesion severity, and the IDV reduced tissue bacterial load, both experimental prototypes were outperformed by the SCV...”

The original version of this article has been updated.

