# Supplementary material for: Evaluation of alternative vaccination routes against paratuberculosis in goats
Source: Front Vet Sci. 2024 Nov 27;11:1457849. doi: 10.3389/fvets.2024.1457849 (PMC11631874; doi:10.3389/fvets.2024.1457849)
Supplement: Supplementary file 1 [file Data_Sheet_1.pdf]

# *Supplementary Material*

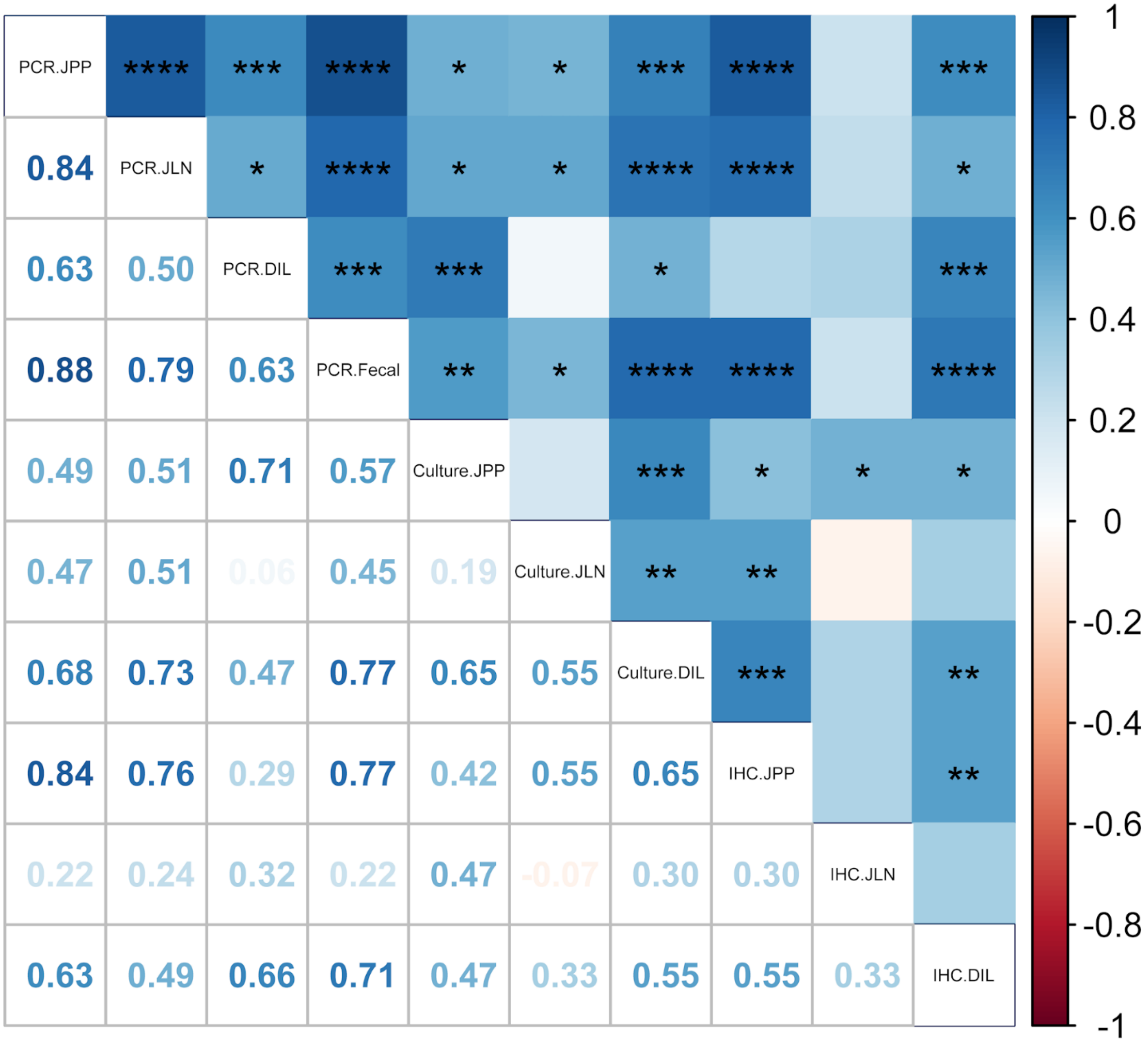


**Supplementary Figure 1. Correlation between the different techniques used for direct *Map* detection.** *Map* DNA quantification in DJPP (PCR.JPP), JLN (PCR.JLN), DIL (PCR.DIL) and 10 MPI fecal samples (PCR.Fecal); *Map* detection through culture in DJPP (Culture.JPP), JLN (Culture.JLN) and DIL (Culture.DIL); *Map* detection through immunohistochemistry in DJPP (IHC.JPP), JLN (IHC.JLN) and DIL (IHC.DIL). DIL (distal ileum), DJPP (distal jejunal Peyer's patch) and JLN (jejunal lymph node). Results are expressed as Spearman's correlation coefficients. Asterisks denote Bonferroni-adjusted $p$ values as follows: * ($p < 0.05$), ** ($p < 0.01$), *** ($p < 0.001$) and **** ($p < 0.0001$).

**Supplementary Table 1. Summary of the statistical analysis on the kinetics of the peripheral immune responses.** [a] Months post-vaccination (mpv) at which the increase in the mean levels was significant ($p < 0.05$) with respect to those at day 0. [b]Month post-vaccination when the highest mean levels were reached and, in parentheses, mean value for that month. Asterisks denote the statistical significance * ($p < 0.05$), ** ($p < 0.01$), *** ($p < 0.001$) and **** ($p < 0.0001$). ND: No detected.

| **Group** | **Increase in *Map*-specific antibody levels[a]** | **Highest *Map*-specific antibody levels reached[b]** | **Increase in AV index[a]** | **Highest AV index reached[b]** | **Increase in BOV index[a]** | **Highest BOV index reached[b]** |
|---|---|---|---|---|---|---|
| **OV** | ND | 9 mpv (0.36) | 6-8 mpv | 7 mpv (2.67) ** | 6-8 mpv | 7 mpv (2.72) *** |
| **OV-INF** | 10 mpv | 11 mpv (4.29) | 5-11 mpv | 9 mpv (3.61) *** | 5-11 mpv | 7 mpv (3.49) *** |
| **IDV** | ND | 10 mpv (0.79) | 6-8 mpv | 7 mpv (2.41) ** | 6-8 mpv | 7 mpv (2.46) ** |
| **IDV-INF** | 5-11 mpv | 9 mpv (2.03) * | 4-11 mpv | 6 mpv (5.17) ** | 4-11 mpv | 4 mpv (4.2) ** |
| **SCV** | 2-11 mpv | 4 mpv (6.69) * | 3-11 mpv | 4 mpv (5.3) *** | 4-11 mpv | 4 mpv (5.08) ** |
| **SCV-INF** | 3-11 mpv | 5 mpv (6.27) ** | 4-11 mpv | 5 mpv (4.71) ** | 3-11 mpv | 4 mpv (3.76) ** |
| **NV** | ND | 9 mpv (0.55) | ND | 5 mpv (1.73) | ND | 5 mpv (1.55) |
| **NV-INF** | 5, 7-11 mpv | 10 mpv (3.16) *** | 4-11 mpv | 6 mpv (4.22) *** | 4-11 mpv | 7 mpv (2.85) *** |

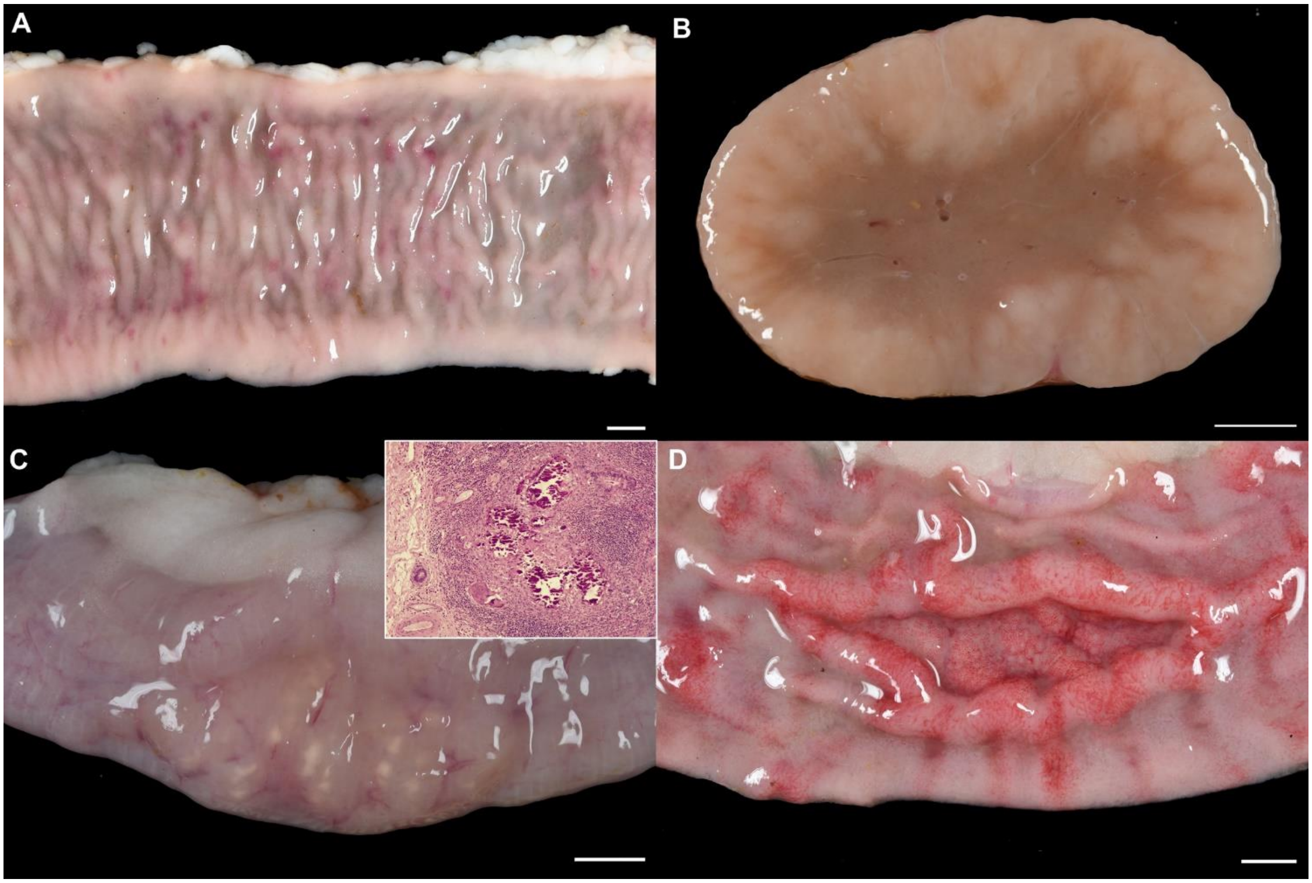


**Supplementary Figure 2. Gross lesions compatible with paratuberculosis.** (A) The intestinal section shows thickened intestinal walls and mucosal folds (Jejunum, Goat 83, NV-INF group). (B) Jejunal lymph node showing cortical enlargement (Jejunal lymph node, Goat 42, IDV-INF group). (C) Intestinal lymphangiectasia, in the Peyer's patch multiple caseous necrosis foci can be observed. Inset: H-E: granulomas with central caseous necrotic foci and mineralization, located in the lymphoid tissue (Jejunal Peyer's patch, Goat 23, OV-INF group). (D) Thickening and congestion of the intestinal mucosa and Peyer's patch (Jejunum, Goat 42, IDV-INF group). Scale bars equal 10 mm.
